# Estimating the Sizes of Populations at High Risk for HIV: A Comparison Study

**DOI:** 10.1371/journal.pone.0095601

**Published:** 2014-04-22

**Authors:** Liwei Jing, Chengyi Qu, Hongmei Yu, Tong Wang, Yuehua Cui

**Affiliations:** 1 Department of Health Statistics, School of Public Health, Shanxi Medical University, Taiyuan, China; 2 Department of Epidemiology, School of Public Health, Shanxi Medical University, Taiyuan, China; 3 Department of Statistics & Probability, Michigan State University, East Lansing, United States; The University of Tokyo, Japan

## Abstract

**Objectives:**

Behavioral interventions are effective strategies for HIV/AIDS prevention and control. However, *implementation* of such strategies relies heavily on the accurate estimation of the high-risk population size. The multiplier method and generalized network scale-up method were recommended to estimate the population size of those at high risk for HIV by UNAIDS/WHO in 2003 and 2010, respectively. This study aims to assess and compare the two methods for estimating the size of populations at high risk for HIV, and to provide practical guidelines and suggestions for implementing the two methods.

**Methods:**

Studies of the multiplier method used to estimate the population prevalence of men who have sex with men in China published between July 1, 2003 and July 1, 2013 were reviewed. The generalized network scale-up method was applied to estimate the population prevalence of men who have sex with men in the urban district of Taiyuan, China.

**Results:**

The median of studies using the multiplier method to estimate the population prevalence of men who have sex with men in China was 4–8 times lower than the national level estimate. Meanwhile, the estimate of the generalized network scale-up method fell within the range of national level estimate.

**Conclusions:**

When high-quality existing data are not readily available, the multiplier method frequently yields underestimated results. We thus suggest that the generalized network scale-up method is preferred when sampling frames for the general population and accurate demographic information are available.

## Introduction

Populations at high risk for human immunodeficiency virus (HIV) mainly include female sex workers (FSW), men who have sex with men (MSM), and injection drug users (IDUs) [1]. These groups are considered as hidden or hard-to-reach populations with two characteristics that create difficulties for estimating population size: 1) They tend to hide their true identities from the public because of the stigma; and 2) There is no sampling frame used for surveys [2]. However, without reliable estimates of the size of these at-risk populations, the ability of governments to carry out intervention planning, resource allocation, estimate the number of people infected with HIV, project disease burden, measure coverage, and evaluate interventions is limited [3]. With these issues in mind, the Joint United Nations Programme on HIV/AIDS (UNAIDS) and the World Health Organization (WHO) released guidelines in 2003, which were updated in 2010, for estimating the size of populations at high risk for HIV [1], [3]. The guideline encompasses census and enumeration methods, capture-recapture method, nomination methods, multiplier method, population surveys and generalized network scale-up methods.

The multiplier method and generalized network scale-up method were used to estimate the number of heavy-drug users in Curitiba, Brazil in 2011 [4]. However, Salganik *et al*. found that the estimate yielded by the generalized network scale-up method was 10 times higher than that of the multiplier method. It is possible that the generalized network scale-up method produced overestimates or the multiplier method produced underestimates in the absence of a gold standard for the number of heavy-drug users. Thus, Salganik *et al*. recommended that additional studies be undertaken to assess these two methods. Here, we compared the multiplier and generalized network scale-up methods by considering previous studies using the multiplier method to estimate the population prevalence of MSM in China and using the generalized network scale-up method to estimate the population prevalence of MSM in Taiyuan, China.

## Materials and Methods

First, studies of the multiplier method used to estimate the population prevalence of MSM in China were reviewed. Second, the generalized network scale-up method was applied to estimate the population prevalence of MSM in the urban district of Taiyuan, China. A national-level estimate proposed by the National Center for AIDS and Sexually Transmitted Disease Control and Prevention in collaboration with UNAIDS/WHO in 2007 was selected as a reference to assess these two results. This estimate was based on high-risk population size surveys, behavioral surveillance surveys, literature searches, and expert estimates. The overall population prevalence of urban MSM in China was estimated to be 2–4% of the male adult population (aged 15–49) [5].

### Multiplier Method

The multiplier method relies on two sources of data [1]. The first source should be high-quality existing data (program data), and the second source a representative survey where members of the high-risk population should be asked whether they received service. The number who received service should then be divided by the proportion reporting having received service in the survey to estimate the size of the populations at high risk for HIV. Two important assumptions must be made: 1) the members of the population must all have a chance of being included in both the survey and the existing data, and 2) the two data sources must be independent, i.e., inclusion in the existing data is not related to inclusion in the survey data [6].

Studies of the multiplier method used to estimate the population prevalence of MSM in China published between July 1, 2003 and July 1, 2013 were reviewed. MOOSE recommendations were used to conduct the reviews [7]. Five electronic databases were searched in Chinese and English: PubMed, Wanfang Data, China National Knowledge Infrastructure, VIP Database for Chinese Technical Periodicals, and Chinese BioMedical Literature Database.

### Generalized Network Scale-up Method

The network scale-up method was first proposed by Bernard, Killworth, Johnsen, and Robinson in 1991 and uses information collected in general population surveys to estimate the size of populations that have a high risk of HIV [8]–[16]. The method is based on the idea that an individual’s personal network reflects the general population in a given region and involves two steps: 1) Estimation of the average personal network size of the general population in a region using the formula *c* = (*m_0_*×*t*)/*e_0_*, where *c* is the average personal network size of the general population, *t* is the total population in the region, *e_0_* is the sum of a list of populations of known size, and *m_0_* is the sum of reported populations in *e_0_* for each respondent from the general population; and 2) Estimation of the size of populations with a high risk of HIV where respondents from the general population are asked how many people they know in the high-risk population. The formula *e_1_* = (*m_1_*/*c*)×*t* is then applied, where *e_1_* is the size of the high-risk population, *m_1_* is the average number of high-risk population members known to respondents from the general population, *c* is the average personal network size of the general population, and *t* is the total population in the region.

An important assumption must be made: respondents from the general population are aware of the high-risk behavior of their acquaintances. Violation of this assumption will lead to information transmission bias [8]–[16]. However, the strength of the generalized network scale-up method proposed by Salganik *et al.* based on the network scale-up method lies in adjusting information transmission bias with the popularity ratio and information transmission rate that can be obtained from information collected from high-risk population surveys [4], [17]. The generalized network scale-up method is carried out in three steps: 1) Estimation of the popularity ratio *δ*, which is the ratio of the average size of a personal network of a high-risk population to the average personal network size of the general population. The average personal network size of the high-risk population can be estimated by applying network scale-up method to the high-risk population itself; 2) Estimation of the information transmission rate *τ*, which is the ratio of acquaintances of the high-risk population who are aware of their high-risk behaviors to all acquaintances of the high-risk population, which can be estimated by interviewing the high-risk population; and 3) Calculation of the adjusted size using the formula *e_2_* = *e_1_*/(*δ*×*τ*), where *e_2_* is the adjusted size of the high-risk population and *e_1_* is a network scale-up estimator.

Most implementations of network scale-up method rely on traditional survey approaches to obtain a representative sample of the population, such as random-digit dial telephone surveys, or face-to-face household surveys where houses are sampled randomly from the equivalent of tracks [8]–[16]. Both telephone interviews and household surveys were used in a pilot survey in our study, but we found that response rates for these methods were less than twenty percent. Suspicion of telemarketing fraud, a common problem in mainland China, is likely a major factor in the low response rate for telephone interviews, while for household surveys, respondents might be embarrassed to admit that they know people with high-risk behaviors due to the serious problem in China of stigma and discrimination against such behaviors, which again may lead to response bias and a low response rate [18], [19]. Therefore, surveys of the general population were carried out in the workplace.

Randomized response technique (RRT) is a survey technique developed by Warner in 1965 that is designed to eliminate response bias when sensitive questions are asked [20]. In 1971 Greenberg introduced the unrelated question RRT model, which allows the interviewer to ask questions requiring a quantitative response [21]. We employed the RRT in a general population survey. Respondents were interviewed in a conference room to provide anonymity and no personal information was collected to ensure genuine answers. Respondents randomly received either a quantitative sensitive question or a quantitative unrelated question. The quantitative sensitive question was: “How many MSM do you know?” “Knowing” was defined as “you know them and they know you by sight or by name, they live in the urban district of Taiyuan, and you have had some contact with them in the past 12 months.” The unrelated question was “How many hours do you spend watching TV per week on average?” The sensitive or unrelated question was printed on the inside wall of an envelope and the interviewer did not know which question as being answered by the respondent. Similarly, other respondents in the room also could not see which question was asked. This question randomization protected the privacy of the respondents. Before starting, we explained to the respondents how RRT works to dispel suspicions and win confidence. Then respondents were divided into two groups randomly which received the first kind of questionnaire (the proportion of sensitive question to unrelated question is *m:n*) and the second kind of questionnaire (the proportion of sensitive question to unrelated question is *n:m*) respectively. Finally, respondents were asked to place their completed questionnaires in a ballot box, which again allowed honest responses to sensitive questions while maintaining confidentiality.

The mean of response to the sensitive question was calculated from the two proportions that the sensitive question was received by the respondent and the two means of responses from the two groups. However, the obtained was only the mean for the department (second-stage unit). Zhu *et al*. explored formulae of the mean and variance for a quantitative sensitive question survey for an unrelated question RRT model in a stratified two-stage cluster sampling in 2009 (see Formula S1), which supports the calculation of the mean and variance of the primary unit and population (*m_1_*) in our survey [22].

## Results

### Results based on Studies of the Multiplier Method

**Figure 1:**
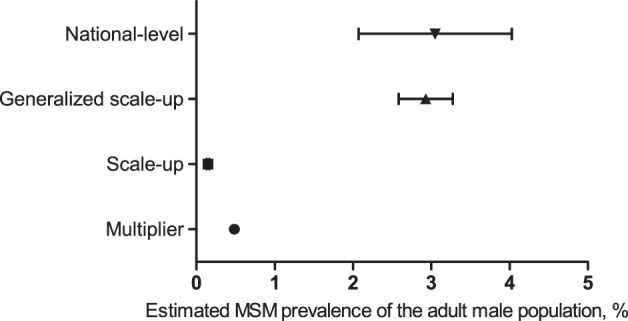
Multiplier estimate and network scale-up estimates compared to the national level estimate.

After the removal of 5 studies (3 duplicate publications and 2 studies that did not obtain appropriate data), 13 studies remained (1 in English and 12 in Chinese) that originated from 13 cities in 11 provinces (including autonomous regions and municipalities) [23]–[33]. The 11 provinces had 7 grades (Table 1) in that all China provinces are divided into 7 grades according to the number of people living with HIV/AIDS (PLHIV) as determined by the Ministry of Health of China, UNAIDS and WHO [34]. Table 1 also shows the city and year of the 13 studies, estimated MSM prevalence of the adult male population (aged 15–49) and the existing data of multiplier method. The estimated MSM prevalence of the adult male population of the 13 cities was 0.066%, 0.249%, 0.486%, 1.220% and 6.884% for the minimum, 25^th^ percentile, median, 75^th^ percentile, and maximum, respectively. The median (0.486%) of the 13 cities was 4–8 times lower than the national-level estimate (2–4%) (Figure 1).

**Table 1:** Multiplier estimates of MSM prevalence in China cities [22]–[33].

City, year	Province	PLHIV in the province	Multiplier estimates of MSM prevalence	The existing data of multiplier method
Chengdu, 2005	Sichuan	80,001–100,000	0.066%	data of MSM visited spots*
Beijing, 2006	Municipality	1,001–5,000	1.000%	data of MSM visited MSM website
Harbin, 2006	Heilongjiang	1,001–5,000	0.300%	data of MSM visited MSM website
Guiyang, 2006	Guizhou	30,001–50,000	0.198%	data of MSM visited MSM website
Hangzhou, 2006	Zhejiang	10,001–30,000	0.150%	data of MSM visited spots
Shanghai, 2007	Municipality	10,001–30,000	6.884%	data of MSM visited MSM website
Wuhan, 2007	Hubei	10,001–30,000	0.486%	data of MSM visited spots
Guangzhou, 2009	Guangdong	50,001–80,000	1.430%	data of MSM visited spots
Yinchuan, 2009	Ningxia	1–1,000	1.200%	data of MSM visited MSM website
Nanjing, 2010	Jiangsu	10,001–30,000	1.240%	data of MSM visited spots and MSM website
Wenzhou, 2010	Zhejiang	10,001–30,000	0.480%	data of MSM visited MSM website
Handan, 2011	Hebei	5,000–10,001	0.335%	data of MSM visitied spots
Jingzhou, 2012	Hubei	10,001–30,000	0.700%	sentinel surveillance

*Spots including bars, public bathhouses, parks, public toilet and internet bar.

### Results Obtained with the Generalized Network Scale-up Method

The generalized network scale-up method was applied to estimate the population prevalence of MSM in the urban district of Taiyuan, China. Taiyuan is the capital city of Shanxi Province (PLHIV grade is 5,000–10,001, which is close to the average grade of all provinces in China) in mainland China and has a total population of 4.23 million residents within an area of 6,959 km^2^, of whom 3.45 million constitute the urban population and occupy 1,460 km^2^. A survey of the general population using stratified two-stage cluster sampling was conducted in the urban district of Taiyuan. For the first stage, 174 primary units (institutions/organizations/companies/governments) from all 20 industries were selected from the sampling frame, with the probability of selection being proportional to industry size. For the second stage, 405 second-stage units (departments) were drawn from those chosen in the first stage wherein 8,031 third-stage units (respondents) aged 18 and above were interviewed in their workplaces, and 231 laid-off workers (unemployed people who still can receive an allowance for family maintenance every month from their former units) were interviewed in their former workplaces as a member of their former industry unit (departments). Respondents of department were divided into two groups randomly. The first kind of questionnaire (the proportion of sensitive question to unrelated question is *8∶2*) was received by the respondents of group 1, while the second kind of questionnaire (the proportion of sensitive question to unrelated question is *2∶8*) was received by the respondents of group 2. A survey of high-risk populations was conducted via respondent-driven sampling (RDS), and 319 MSM were interviewed [35], [36]. All of the above-mentioned surveys were carried out in March 2012 and October 2012.

**Figure 2:**
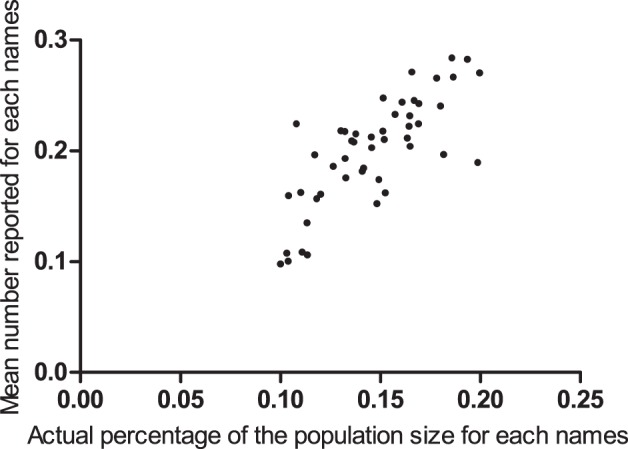
Reported number compared to the actual percentage of the population size.

We estimated the average personal network size of the general population and MSM by asking: “How many people (acquaintances) do you know whose last names are on a list of 48 last names (e.g. Yi, Chang, Pang, Lan, An, Niu, Shen, Xing, Mei, and Mo)?” The definition of “knowing” was as indicated in the Methods section. Each of the 48 last names accounted for 0.1–0.2% of the urban population and these last names were selected to minimize recall bias [37]. The demographic information was provided by the Taiyuan Public Security Bureau (see Table S1). The procedure worked well in that the mean number reported for each of the 48 known-size last names strongly correlated with the actual percentage of the population size for each of the 48 last names (*r* = 0.771, *P*<0.01) (Figure 2) The maximum-likelihood estimate of the average personal network size for the general population was 137 and 145 (*δ* = 1.06) for MSM. The survey of MSM was conducted via RDS, and a bootstrap method was used to calculate the confidence interval of *δ* (95% CI: 0.81, 1.42) [4], [17].

We interviewed MSM by asking: “How many people (acquaintances) with the last name Yi are aware of your high-risk behavior?”, “How many people (acquaintances) with the last name Chang are aware of your high-risk behavior?”, until each of the 48 last names was covered. The reported number for the question “How many acquaintances are aware of your high-risk behavior?” by MSM was very low, which indicates that very few people are aware of the true sexuality of MSM. Thus, the estimated information transmission rate of MSM in Taiyuan was only 4.8%. Due to influences of traditional Chinese culture, people have low tolerance for MSM in mainland China [18], [19]. Thus, because of the potential for stigma and discrimination, MSM must avoid disclosure. The survey of MSM was conducted via RDS, and a bootstrap method was used to calculate the confidence interval of information transmission rate (95% confidence interval (CI): 0.0%, 10.2%) [4], [17].

The estimate *e_1_* of the network scale-up method of MSM in the urban district of Taiyuan was 1,367 (95% CI: 1,199, 1,536), which corresponds to 0.149% (95% CI: 0.131%, 0.167%) of the adult male population (aged 15–49) (Figure 1). The lower limit of 95% CI of *m_1_* (average number of MSM members known to respondents from the general population) was used to estimate the lower limit of 95% CI of *e_1_* (network scale-up estimator) using the formula *e_1_* = (*m_1_*/*c*)×*t*, and the upper limit of 95% CI of *m_1_* was used to estimate the upper limit of 95% CI of *e_1_* in the same way.

The generalized network scale-up method allowed us to estimate the number of MSM in Taiyuan as being 26,870 (95% CI: 23,556, 30,184), which corresponds to 2.927% (95% CI: 2.566%, 3.288%) of the adult male population (aged 15–49). The lower and upper limit of 95% CI of *e_1_* were used to estimate the lower and upper limit of 95% CI of *e_2_* (generalized network scale-up estimator) using the formula *e_2_* = *e_1_*/(*δ*×*τ*). By adjusting information transmission bias with the popularity ratio and the information transmission rate, the estimate of the generalized network scale-up method falls within the range of national-level estimates (2–4%) (Figure 1).

## Discussion

The generalized network scale-up method is based mainly on data collected from the general population. Respondents might be highly reluctant to admit that they know people with high-risk behaviors, which lead to response bias. As such, protection of privacy is the basis for reliable results. For the first time, a generalized network scale-up method combined with RRT was applied to estimate the size of populations at high risk for HIV, and 96.4% of all survey respondents were successfully interviewed.

The generalized network scale-up method and multiplier method differed greatly in their estimates for drug users in Brazil largely due to assumption violations of multiplier method that produced underestimates. Upper-middle-class drug users were less likely to participate in both drug treatment programs offered by local governments (the existing data) and the RDS survey (the survey data) [4]. The second assumption of independence was violated because inclusion in the existing data was related to inclusion in the survey data. As a result, lower-class drug users were more likely to participate in both drug treatment programs and the RDS survey; therefore, excessive overlap of the two data sources led to underestimation. Similarly, study reviews showed that the multiplier estimate of the MSM population in China produced underestimates mainly because the assumptions regarding MSM as estimated by the multiplier method were not readily met [23]–[33]. Specifically, except for Jingzhou in MSM studies, the entire existing data for 12 cities were gathered from websites or places such as bars, public bathhouses, parks, public toilets, and internet bars (Table 1). Such collection sites can be problematic because: 1) not every MSM is likely to visit such locations or websites, and thus selection bias cannot be avoided; 2) the number of MSM attending such locations within a certain time period was recorded by researchers with the help of an insider such that MSM were identified by their appearance, figure, and behavior. However, members of the MSM population who never manifested the above-mentioned characteristics would not be included in the existing data, but could still be included in survey data because of the RDS survey. Thus, this situation led to underestimation; and 3) the number of MSM visiting the MSM website within a certain time period was recorded by the website based on IP addresses. If two MSM visited the MSM website using the same IP address, one of them would not be included in the existing data but could still be included in the survey data. This situation also led to underestimation. In addition, the existing data for Jingzhou were obtained by sentinel surveillance, but the manner in which the survey data were acquired was very similar to that used to gather the existing data. The second assumption of independence was violated because inclusion in the existing data was related to inclusion in the survey data. As a result, excessive overlap between the two data source led to underestimation.

## Conclusions

Compared with the generalized network scale-up method, the most important advantage of the multiplier method is that it is much more straightforward to use. However, multiplier estimates depend on high-quality existing data that ensures that each individual member of the high-risk population has a chance of being included. Because such high-quality existing data are often unavailable, the multiplier method has frequently been misused. Therefore, we suggest that a high-quality existing data should have the key characteristic that each individual in the high-risk population has a chance to be included in the existing data because of needing help. For instance, each female sex worker (FSW) is likely to be infected with any sexually transmitted diseases (STD), moreover almost each infected FSW would seek medical attention because of needing help, thereby data of STD clinics is a high-quality existing data. Further, identities are readily determined because of needing help. For instance, FSW would admit their identities in STD clinics, because it could help for disease diagnosis. Thus, the number of FSW who attended the STD clinics can be recorded accurately.

Although the generalized network scale-up method has not been widely applied in the field of epidemiology, it is still a promising approach that has three main advantages: 1) the method is based on data collected from the general population and a small sample from high-risk populations; 2) respondents are often more likely to report on the high-risk behavior of others rather than their own high-risk behavior; and 3) a single survey can yield estimates for multiple populations having a high risk for HIV [1]. We suggest that the generalized network scale-up method is preferred when sampling frames for the general population and accurate demographic information are available.

## Limitation

In this study, respondents randomly received either a sensitive question or a unrelated question, therefore which question was received and responded by them were not known. Although the privacy of the respondents was protected in this situation, it brought new problems. Variance estimation of *e_1_* and *e_2_* has not fully worked out in theory, thus we could not estimate them at present. The lower and upper limits of 95% CI of *m_1_* were simply used to estimate the lower and upper limit of 95% CI of *e_1_*. Then the lower and upper limits of 95% CI of *e_1_* were used to estimate the lower and upper limit of 95% CI of *e_2_* in the same way. Such confidence intervals are almost always less than true confidence intervals when complex sampling was applied in the survey and more innovative work are needed in the future.

## Supporting Information

Table S1 The list of 48 last names.
(DOC)

Formula S1 (DOC)
