# Supplementary material for: Estimating the Sizes of Populations at High Risk for HIV: A Comparison Study
Source: PLoS One. 2014 Apr 22;9(4):e95601. doi: 10.1371/journal.pone.0095601 (PMC3995743; doi:10.1371/journal.pone.0095601)
Supplement: Table S1 — The list of 48 last names. (DOC) [file pone.0095601.s001.doc]

**Table S1.** The list of 48 last names.

| **English** | **China** | **Population size** | **Proportion of the urban population** |
| --- | --- | --- | --- |
| Yan | 沿 | 3455 | 0.001000 |
| Cong | 从 | 3567 | 0.001032 |
| Wu | 伍 | 3585 | 0.001038 |
| Zhang | 章 | 3596 | 0.001041 |
| Tang | 汤 | 3730 | 0.001080 |
| Mei | 梅 | 3807 | 0.001102 |
| Yi | 易 | 3831 | 0.001109 |
| Xiang | 向 | 3913 | 0.001133 |
| You | 尤 | 3925 | 0.001136 |
| Shi | 施 | 4048 | 0.001172 |
| Li | 黎 | 4081 | 0.001181 |
| Yu | 俞 | 4148 | 0.001201 |
| Ni | 倪 | 4370 | 0.001265 |
| Hong | 洪 | 4501 | 0.001303 |
| Yan | 颜 | 4572 | 0.001323 |
| Mo | 莫 | 4576 | 0.001324 |
| Lu | 鲁 | 4584 | 0.001327 |
| Lu | 芦 | 4688 | 0.001357 |
| Wei | 韦 | 4723 | 0.001367 |
| Yin | 殷 | 4759 | 0.001377 |
| Gong | 龚 | 4867 | 0.001409 |
| Ji | 季 | 4890 | 0.001415 |
| Lan | 兰 | 5024 | 0.001454 |
| Bi | 毕 | 5029 | 0.001456 |
| Qi | 齐 | 5121 | 0.001482 |
| Tao | 陶 | 5156 | 0.001492 |
| Lu | 路 | 5225 | 0.001512 |
| Wen | 文 | 5235 | 0.001515 |
| Yan | 严 | 5249 | 0.001519 |
| Liu | 柳 | 5266 | 0.001524 |
| Lai | 赖 | 5440 | 0.001575 |
| Yue | 岳 | 5561 | 0.001610 |
| Pang | 庞 | 5644 | 0.001634 |
| Ge | 葛 | 5681 | 0.001644 |
| Wen | 温 | 5689 | 0.001647 |
| Nie | 聂 | 5700 | 0.001650 |
| Shen | 申 | 5726 | 0.001657 |
| Yin | 尹 | 5764 | 0.001668 |
| Jiao | 焦 | 5841 | 0.001691 |
| Xing | 邢 | 5847 | 0.001692 |
| An | 安 | 6155 | 0.001782 |
| Zhai | 翟 | 6220 | 0.001800 |
| He | 贺 | 6273 | 0.001816 |
| Fan | 樊 | 6417 | 0.001857 |
| Chang | 常 | 6441 | 0.001864 |
| Niu | 牛 | 6687 | 0.001936 |
| Qiao | 乔 | 6864 | 0.001987 |
| Wu | 武 | 6897 | 0.001996 |
