# Supplementary material for: Estimating the Sizes of Populations at High Risk for HIV: A Comparison Study
Source: PLoS One. 2014 Apr 22;9(4):e95601. doi: 10.1371/journal.pone.0095601 (PMC3995743; doi:10.1371/journal.pone.0095601)
Supplement: Formula S1 — (DOC) [file pone.0095601.s002.doc]

Suppose that the population is composed of *L* industries. The *h*th industry contains *N*_1_*_h_* primary units. The *i*th primary unit of the *h*th industry contains *N_i_*_2_*_h_* second stage units. In average, each primary unit of the *h*th industry contains $\overline{N}_{2h}$ second-stage units. The *j*th second stage unit of the *i*th primary unit of the *h*th industry contains *N_ij_*_3_*_h_* third-stage units. In average, each second stage of the *h*th industry contains $\overline{N}_{3h}$ third-stage units. The population totally contains *N* third-stage units.

For the first stage, *n*_1_*_h_* primary units from the *h*th industry were selected from the sampling frame, with the probability of selection being proportional to industry size. For the second stage, *n_i_*_2_*_h_* second-stage units were chosen from the *i*th chosen primary unit of the *h*th industry. In average, $\overline{n}_{2h}$ second-stage units were chosen from each chosen primary unit of the *h*th industry. All the third-stage units were interviewed from the chosen second stage unit. The third-stage units of each second stage were divided into two groups randomly. The first kind of questionnaire (the proportion of sensitive question to unrelated question is *m:n*) was received by the third-stage units of group 1, while the second kind of questionnaire (the proportion of sensitive question to unrelated question is *n:m*) was received by the third-stage units of group 2.

The mean obtained for the *j*th second-stage unit drawn from the *i*th primary unit of the *h*th industry is formula (1).

$\mu_{ijh}=\frac{\left(1-P_{2}\right)\times\mu_{ijh1}-\left(1-P_{1}\right)\times\mu_{ijh2}}{P_{1}-P_{2}}$ (1)

Where *μ_ijh_*_1_ is the mean of responses from group 1, *μ_ijh_*_2_ is the mean of responses from group 2, *P_1_* is *m/(m+n)*, i.e. the proportion of sensitive question in the first kind of questionnaire, *P_2_* is *n/(m+n)*, i.e. the proportion of sensitive question in the second kind of questionnaire.

The estimator of the mean of the *i*th primary unit drawn from the *h*th industry is formula (2).

$\hat{\mu}_{ih}=\frac{\sum_{j=1}^{n_{i2h}} N_{ij3h}\mu_{ijh}}{\sum_{j=1}^{n_{i2h}} N_{ij3h}}$ (2)

The estimator of the mean of the *h*th industry is formula (3).

$\hat{\mu}_{h}=\frac{N_{1h}}{N_{h}n_{1h}}\sum_{i=1}^{n_{1h}} \frac{N_{i2h}}{n_{i2h}}\sum_{j=1}^{n_{i2h}} N_{ij3h}\mu_{ijh}$ (3)

The variance of $\hat{\mu}_{h}$ is formula (4).

$\upsilon\left(\hat{\mu}_{h}\right)=\frac{S_{1h}^{2}}{n_{1h}}\left(1-\frac{n_{1h}}{N_{1h}}\right)+\frac{S_{2h}^{2}}{n_{1h}\bar{n}_{2h}}\left(1-\frac{\bar{n}_{2h}}{\bar{N}_{2h}}\right)$ (4)

$$S_{1h}^{2}=\frac{1}{n_{1h}-1}\sum_{i=1}^{n_{1h}} \left(\hat{\mu}_{ih}-\hat{\mu}_{h}\right)^{2}$$

$$S_{2h}^{2}=\frac{1}{n_{1h}}\sum_{i=1}^{n_{1h}} \frac{1}{n_{i2h}-1}\sum_{j=1}^{n_{i2h}} \left(\mu_{ijh}-\hat{\mu}_{ih}\right)^{2}$$

The estimator of the population mean is formula (5).

$\hat{\mu}=\sum_{h=1}^{L} \frac{N_{h}}{N}\frac{N_{1h}}{N_{h}n_{1h}}\sum_{i=1}^{n_{1h}} \frac{N_{i2h}}{n_{i2h}}\sum_{j=1}^{n_{i2h}} N_{ij3h}\mu_{ijh}$ (5)

The variance of $\hat{\mu}$ is formula (6).

$\upsilon\left(\hat{\mu}\right)=\sum_{i=1}^{L} \left(\frac{N_{h}}{N}\right)^{2}\left[\frac{S_{1h}^{2}}{n_{1h}}\left(1-\frac{n_{1h}}{N_{1h}}\right)+\frac{S_{2h}^{2}}{n_{1h}\bar{n}_{2h}}\left(1-\frac{\bar{n}_{2h}}{\bar{N}_{2h}}\right)\right]$ (6)

The 95% confidence interval $\hat{\mu}$ is formula (7).

$\hat{\mu}\pm1.96\times\sqrt{\upsilon\left(\hat{\mu}\right)}$ (7)
